# Postgenomic understandings of fatness and metabolism

**DOI:** 10.1007/s40656-024-00630-w

**Published:** 2024-10-30

**Authors:** Azita Chellappoo

**Affiliations:** The Open University, Walton Hall, Kents Hill, Milton Keynes, MK7 6AA UK

**Keywords:** Metabolism, Postgenomics, Determinism, Environment, Obesity

## Abstract

‘Obesity’ has, for decades, been a subject of intense scientific and public interest, and remains a key target for postgenomic science. I examine the emergence of determinism in research into ‘obesity’ in the postgenomic field of metabolomics. I argue that determinism appears in metabolomics research in two ways: firstly, fragmentation and narrow construal of the environment is evident in metabolomics studies on weight loss interventions, resulting in particular features of the environment (notably, dietary intake) having outsized influence while the wider social environment is neglected. Secondly, studies aiming to characterize the metabolic signature of ‘obesity’ are guided by a commitment to a deterministic connection between ‘obesity’ and dysfunction, leading to a neglect or distortion of metabolic heterogeneity across individuals regardless of body size.

## Introduction

‘Obesity’ has been a target of significant interest, both for scientists and the public, for decades. ‘Obesity’ as a problem to be solved garnered some attention in countries such as the UK and the US in the 1970s and 1980s in the form of government reports and the creation of research centres (James, 2008). However, it was not until the 1990s that ‘obesity’ came to be considered a critical public health issue, with intensified focus on the supposed negative health and economic effects of ‘obesity’ (Lupton, 2018). ‘Obesity’ began to be seen as a problem affecting people around the world (James, 2008). The International Obesity Task Force was established in 1996, with the purpose of addressing the “emerging global epidemic of obesity” (IOTF, 2002). Along with attention from governments, clinicians, researchers, and health organisations, ‘obesity’ became an attention-grabbing topic for media outlets. In the 2000s, New York Times headlines included “As America Gets Bigger, the World Does, Too”, and “America’s Epidemic of Youth Obesity”, and Time magazine dedicated covers to the issue, with titles of “Overcoming Obesity in America” and “Our Super Sized Kids”.

The characterization of ‘obesity’ as an ‘epidemic’ in media and scientific articles invokes an analogy to the spread of infectious diseases to refer to the claim that rates of ‘obesity’ are rising rapidly (Gilman, 2008). The ‘obesity epidemic’ remains a frequently used term in media and communications from international health organisations (World Health Organisation, 2022). In the scientific and public health literature, ‘obesity’ is sometimes upgraded from ‘epidemic’ to a ‘pandemic’ which is sweeping the globe (Roth et al., 2004; Spanier et al., 2006; Swinburn et al., 2011). During the Covid-19 pandemic, ‘obesity’ received renewed attention as a potential contributing factor to the health burden of Covid-19, and the interaction between ‘obesity’ and Covid-19 was termed a ‘syndemic’ (Hill et al., 2021; Pryor & Dietz, 2022).

The framing of ‘obesity’ as an epidemic or pandemic has helped to justify its position as a key target for public health and clinical intervention, as well as spurring biomedical research to uncover the causes of ‘obesity’ with the goal of developing effective interventions. Various narratives about the causes of ‘obesity’ have emerged over time, including the role of genes, as well as ‘obesity’ as a result of a failure to balance calorie intake against calorie expenditure. The failure to regulate calorie intake and expenditure has been variously attributed to individual weakness of will or psychological pathology (Gilman, 2008), or features of the environment that encourage increased intake and decreased expenditure (Swinburn, 1999; Kirk et al., 2010).

Mainstream scientific and public understandings of ‘obesity’ have not gone without challenge. Biomedical conceptions of ‘obesity’, and their often deterministic assumptions, have been a subject of increasing contestation. The growing interdisciplinary field of fat studies, as well as activists working towards fat justice or fat liberation, have rejected claims that ‘obesity’ is a disease, that there is a clear or deterministic causal relationship between body size and health risk, and that long term weight loss is practical, achievable, and without harm (Rothblum & Solovay, 2009; Campos, 2004; Pausé & Taylor, 2021). These scholars and activists reject the term ‘obesity’ as medicalising and pathologising, preferring the term ‘fat’ as a descriptor (Wann, 2009). In addition, anthropologists and sociologists have cast ‘obesity’ rhetoric and policy as a ‘moral panic’, and drawn attention to the ways in which ‘obesity’ discourses are medicalized, racialised, class-coded, and place responsibility on individuals (Yates-Doerr, 2015; Campos et al., 2006; Saguy & Almeling, 2008; Strings, 2019). In contrast to a biomedical account of ‘obesity’ as either a disease state or a biological state that puts an individual at a higher risk of disease, fat studies scholars and fat justice activists have reframed fatness as a social category, where pervasive structural and interpersonal anti-fat discrimination or weight stigma play a key role (Pausé et al., 2021 provides one example of this reconceptualisation of fatness in the context of Covid-19).

Despite increasing opposition to a biomedical account of ‘obesity’, it remains a key target in contemporary postgenomic science, in fields such as environmental epigenetics, microbiome science, developmental origins of health and disease (DOHaD), and metabolomics. Postgenomic science has been defined both temporally and technically, in terms of the period following the sequencing of the human genome, and in terms of the use of whole-genome technologies in many fields within the biosciences (Stevens & Richardson, 2015). However, scholars have also identified important conceptual shifts associated with postgenomics, including, importantly, a departure from gene-centrism and genetic reductionism (ibid.). Research within fields such as epigenetics, microbiome science, and developmental biology appear to allow for complex entanglements between the social and the biological (Meloni, 2014; Darling et al., 2016; van Wichelen, 2022). The potential for an increasing appreciation of complexity notwithstanding, forms of environmental determinism nevertheless appear to be emerging in postgenomics (Lock, 2013a; Mansfield, 2012; Warin et al., 2020; van Wichelen & Keaney, 2022).

Scholars have explored aspects of ‘obesity’ in postgenomic science (Richardson and Stevens, 2015; Valdez, 2021; Saldaña-Tejeda, 2018; Saldaña-Tejeda & Wade, 2019), particularly in the context of epigenetics and DOHaD research. However, the field of metabolomics has been relatively neglected, both in terms of the study of ‘obesity’ research and more general investigations of postgenomic science. Despite this, developments within metabolomics carry important implications for understandings of metabolism and ‘obesity’ that deserve attention. In addition, current literature on ‘obesity’ in postgenomics does not tend to explore the conceptualisation of ‘obesity’ itself, or the underlying assumptions about ‘obesity’ that structure postgenomic research in this area. In this paper I contribute to addressing both these lacunae. I argue that forms of determinism can be identified within investigations of ‘obesity’ in metabolomics: determinism appears in the fragmentation and narrow construal of the environment in metabolomics studies on weight loss interventions, and in the commitment to a deterministic connection between ‘obesity’ and dysfunction in studies aiming to characterize the metabolic signature of ‘obesity’. This philosophical analysis contributes to the understanding of the forms of determinism emergent within postgenomic science, as well as drawing attention to the ways in which particular challenges arise in the context of the scientific investigation of ‘obesity’.

In Sect. 2 I outline genetic determinism and postgenomic determinism, and their uptake and normative implications in the context of ‘obesity’. In Sect. 3 I introduce the field of metabolomics and its potential for opening up dynamic and interactive understandings of metabolism. Despite this potential, an examination of metabolomic inquiry reveals deterministic trends. In Sect. 4 I argue that environmental determinism appears in metabolomics through the narrow and fragmented conception of the environment reflected in the choice of interventions under study, and the neglect of wider social environmental factors (particularly weight stigma and weight discrimination). In Sect. 5 I argue for the existence of another form of determinism within metabolomics: commitment to the deterministic relationship between ‘obesity’ and dysfunction. I highlight the ways in which this deterministic commitment is evident in studies of the metabolic signature of ‘obesity’ through strategic neglect of metabolic heterogeneity, flexible use of the meaning of ‘obesity’ when considering metabolic heterogeneity, and in how metabolic heterogeneity is classified. I conclude in Sect. 6.

## Determinism and ‘obesity’ in genetics and postgenomics

### Genetic determinism

Genetic determinism, the assertion that genes control or determine a trait, has been both influential and controversial throughout the 20th and 21st Century. Genetic determinism has been subjected to a range of critiques on both epistemic and ethical grounds, including that genetic determinist thinking ignores organismal plasticity and interactions between the genetic and non-genetic factors, that it relies on an impoverished or mistaken understanding of development, and that genetic determinism plays a fundamental role in harmful ideologies such as eugenics (Jablonka, 2012; Lewontin, 1983; Griffiths & Gray, 1994; Roll-Hansen, 2010). Despite the volley of challenges, genetic determinism persists in science education and the public consciousness (Jamieson & Radick, 2017; Shostak et al., 2009), leading Kitcher (2001) to refer to resisting genetic determinism as “battling the undead”. The endurance of popular narratives of genetic determinism can be seen clearly in the case of purported genetic differences between races. Despite ample evidence and arguments against the existence of genetically differentiated races and the causal role of genes in determining so-called racial traits (Graves & Goodman, 2021 provides one overview), genetic determinism about race remains present in public attitudes and media coverage (Hochschild & Sen, 2015; Kowal & Frederic, 2012; Herd et al., 2021).

Despite an ongoing research programme into the genetic causes of ‘obesity’, determinist narratives in this area have been relatively absent. By this I mean that genes are commonly not understood to control, determine, or be primarily causally responsible for ‘obesity’, in scientific literature or public health communications^1^. While the role of genes is acknowledged, genes are often not taken to be primary causal drivers of ‘obesity’.

For example, the multifactorial nature of ‘obesity’ is commonly acknowledged in scientific literature about genetics and ‘obesity’:

With few exceptions, obesity is a complex multifactorial disease. As for other complex human diseases, the identification of genes and mutations causing obesity in a small number of cases does not directly address genetic causes in the population as a whole, but can illuminate candidate pathways involved in the pathophysiology of obesity. Loos & Bouchard, 2003, p. 404.

Additionally, genetic determinist narratives have had limited purchase in public health messaging and wider public discourse. For example, under the heading of ‘genetics’ on the United Kingdom’s National Health Service webpage (NHS, 2019) on the causes of ‘obesity’, the text reads:

Some people claim there’s no point trying to lose weight because “it runs in my family” or “it’s in my genes”. While there are some rare genetic conditions that can cause obesity, such as Prader-Willi syndrome, there’s no reason why most people cannot lose weight.

The absence of genetic determinist narratives about ‘obesity’ have also been identified in media coverage. Caulfield et al. (2009, p39) found a “noticeable shift away from a deterministic framing of obesity” in newspaper coverage from 1999 onwards. Rather, ‘obesity’ came to be portrayed as a matter of lifestyle choices, for which individuals are personally responsible, not “unchangeable environmental or genetic factors”. Additionally, causal attributions of ‘obesity’ to genetic factors by the public appear to be low (Hilbert et al., 2007).

A potential reason for the contrast between the persistence of genetic determinism in the case of race and its relative absence in the case of ‘obesity’ is the landscapes of responsibility and intervention that genetic determinist narratives open up or close off. In the case of race, genetic determinist narratives are deployed to naturalise social inequality and provide justification for the persistence of racial hierarchy. In the case of fatness, the attribution of larger body size to genetic causes is in tension with the attribution of personal responsibility. Studies have shown that individuals who are presented with or endorse causal claims about genetics and ‘obesity’ are more likely to view ‘obesity’ as less controllable and therefore less likely to attribute responsibility for larger body size to individuals or display stigmatizing attitudes towards fat people (Jeong, 2007; Hilbert et al., 2008).

This suggests that the effects or harms of genetic determinist narratives may bear some key differences when it comes to attitudes to fatness in comparison to other kinds of social difference, such as race or gender.^2^ Personal responsibility is a key component of the stigmatising attitudes towards fat people and the maintenance of anti-fat discrimination both interpersonally and structurally, and the attribution of responsibility may be mediated by perceptions of the capacity for individual control. To the extent that genetic determinist narratives imply a lack of individual control over body size, they may reduce stigma or blame. Furthermore, the lack of individual control over body size due to genetic causes plays a role in arguments for the injustice of anti-fat discrimination, such as in Eller’s (2014) account of fat oppression. Eller’s argument proceeds from the claim that inequalities are unjust only when they exist in virtue of a trait assigned by the ‘natural lottery’, and that body size is such a trait, as body size is determined by genes and the environment. The difference in uptake, deployment, and effect of deterministic claims is something to consider when evaluating deterministic narratives arising within postgenomic science.

### Postgenomic determinism

Postgenomic science can be understood as referring to the technological and conceptual developments in the life sciences that have occurred since the completion of the Human Genome Project. The advent of technologies that allow the sequencing not only of whole genomes, but also of RNA transcripts, proteins, and metabolites, appears to have ushered in significant conceptual shifts. This shift has been characterized in terms of a move away from gene-centrism, genetic determinism, and simplistic understandings of the gene as a causal driver, towards a focus on complexity, interactions between genes, organismal physiology, development, and the environment, and a reconceptualization of the gene itself as constituted by such interactions (Richardson & Stevens, 2015; Stotz et al., 2006). Aspects of this shift can be seen in various subdisciplines of the life sciences. Research within environmental epigenetics has illuminated pathways through which the environment can affect patterns of gene expression, in ways that could be passed down across generations. Epigenetic mechanisms present a challenge to the gene as the sole bearer of heredity, and the concept of the atomized gene separable from regulatory interaction (Meloni, 2015; Jablonka & Raz, 2009), as well as producing a picture of the body as imprinted by the past and ‘embedded’ in its social and physical environment (Niewöhner, 2011). Within the developmental origins of health and disease (DOHaD) framework researchers investigate links between health states throughout the life course and *in utero* or early life environmental exposures, often via epigenetic mechanisms. The DOHaD framework attends to interactions between environment, development, and genetics, with a focus on the capacity for plasticity (Gluckman et al., 2010). There has also been increasing attention paid to the significance of the microbiome, the community of microorganisms that reside within an individual, such as those bacteria, archaea, fungi, and viruses that inhabit the human gut. The microbiome plays a role in various aspects of human physiology, including digestion and the immune system, and appreciation of the critical roles it plays troubles the notion of the clearly demarcated biological individual and focuses attention on interactions within a multispecies ecological community (Chiu & Gilbert, 2015; Skillings, 2016).

Postgenomic science and its subdisciplines, with the focus on interactivity and plasticity, offer an apparent alternative to genetic determinism. However, scholars have drawn attention to the ways in which other forms of determinism arise within postgenomics. Richardson (2015), in her analysis of research on maternal-fetal epigenetic programming, describes this research as offering “an expanded but still reductionist and determinate model of development – a “somatic determinism”” (p. 217). This ‘somatic determinism’ emerges from a model wherein the social and physical environment are heritable and determinative of future health outcomes (via the maternal body as critical vector). Others have similarly pointed to emerging environmental (or somatic) determinism, where environmental influences translated into modified gene expression patterns are understood as determinative of traits or outcomes, particularly when these influences are understood to be inherited across generations (Lock, 2013a, b; Waggoner & Uller, 2015). Furthermore, it is not the environment as a whole that is taken to have a determinative role, but rather fragmented or isolated features of the environment that become ‘molecularised’, or reduced to their molecular effects (Niewöhner, 2011; Lock, 2015; further discussion of fragmented environments in postgenomics continues in Sect. 3).

The harmful potential of postgenomic determinism has also been raised, as public uptake of knowledge of postgenomic science such as epigenetics increases. While postgenomic science poses a challenge to genetic determinism and acknowledges the role of the social environment, the environmental determinism emergent in postgenomics may nevertheless reinforce claims about biological racial or sex differences or support essentialism about race or sex/gender (Meloni, 2017; Meloni et al., 2022; Warin et al., 2020; Richardson, 2017; Benezra, 2020). Furthermore, the idea coming out of epigenetics and DOHaD research that pre-conception and early life environments can have long-lasting effects could mark out certain populations (particularly in terms of race and class) as biologically disadvantaged (Mansfield, 2012; Richardson, 2017).

Does determinism arise in postgenomic investigations of ‘obesity’? ‘Obesity’ remains a key target for postgenomic science, and environmental determinism may be emerging within these areas of research. DOHaD researchers have suggested that maternal ‘obesity’ and nutritional exposures in utero could influence the ‘risk of obesity’ later in life, through epigenetic modifications (Van Dijk et al., 2015; Bianco-Motto et al., 2017). Wells (2016) suggests that poor quality maternal environments could trap individuals in “metabolic ghettos”, resulting in ‘obesity’ and metabolic diseases. Microbiome researchers have aimed to characterise the ‘obesogenic’ microbiome, and attempted to identify differences in composition between ‘obese’ and ‘lean’ microbiomes (Bäckhed et al., 2004; Turnbaugh et al., 2009). In this research, the presence of particular microbial taxa play a key causal role in producing ‘obese’ bodies.

The focus in postgenomic research on the role of the maternal environment in determining body size later in life has drawn critiques for the way in which this places the burden of individual responsibility on mothers for preventing ‘obesity’ in their children, potentially reaching back across the pre-conception period (Richardson, 2021; Sharp et al., 2018; Kenney & Muller, 2017). Additionally, the ‘maternal environment’ is construed narrowly in terms of individual behaviour or individual environment, rather than being connected to the broader social environment and patterns of structural advantage and disadvantage, with particular burden or stigma being placed on racialized groups (Warin, 2012; Valdez, 2021; Saldaña-Tejeda, & Wade, 2019). In these cases, postgenomic understandings of ‘obesity’ are deterministic in that the maternal environment is taken to determine foetal ‘obesity’ risk, although individual responsibility and the obligation to make certain choices is still present, becoming shifted onto the mother. On the other hand, some scholars have suggested the conception of bodies as products of complex and entangled biological and social processes found within the DOHaD framework, as well as the connection DOHaD draws between social and economic inequalities and ‘obesity’, could provide a path away from responsibilising or stigmatising discourses (Warin et al., 2016; Warin, 2015; Yoshizawa, 2012).

As with genetic determinism, environmental determinism is in tension with placing responsibility and blame on individuals themselves for their traits (although it is compatible with blaming others, such as mothers). To the extent that environmental determinist narratives about ‘obesity’ arise, they may reduce (or accompany the reduction of) stigmatising and blaming attitudes towards fat individuals. Robison’s (2015, 2016) analysis of narratives of ‘obesity’ and epigenetics in newspaper coverage suggests that, so far, epigenetic narratives about ‘obesity’ don’t show clear tendencies towards environmental determinism: they are sometimes associated with discussions of environmental causes, although they are also sometimes associated with discussions of lifestyle choices which can feed into framings of ‘obesity’ as a matter of personal responsibility.

Environmental determinism, the attribution of control or causal primacy to environmental causes in determining traits, is one form of determinism that is present in postgenomic science. However, in the investigation of ‘obesity’ or larger bodies, other forms of deterministic thinking may also arise. One form of determinism is present in the ‘calories in-calories out’ model, which supposes that body size is solely or overwhelmingly determined by the balance of calorie intake to calorie expenditure. Another form of determinism arises in the assumption that ‘obesity’ is inherently unhealthy, pathological, or a disease: here, body size is taken to be determinative of health outcomes. Neither of these sorts of deterministic models are unique to postgenomic science – they have pre-dated its advent. Nevertheless, they continue to shape postgenomic investigations of ‘obesity’, with implications for how postgenomic research understands and seeks to intervene on fat bodies. In the following section I outline shifts in scientific understandings of metabolism and current research in the postgenomic field of metabolomics in relation to ‘obesity’. I then argue that two forms of determinism appear in metabolomics research into ‘obesity’: environmental determinism in the form of constrained and fragmented features of the environment that are taken to determine metabolic outcomes, and the commitment to body size as determinative of health.

## Metabolic determinism

Understandings of metabolism are intertwined with understandings of ‘obesity’. ‘Obesity’ is often taken to be a cause of or risk factor for metabolic disorders. Additionally, under one dominant conception of variation in body size, the ‘calories-in-calories out’ model, ‘obesity’ is a failure to adequately achieve metabolic balance. Under this model, body size is determined by the calculation of calorie expenditure subtracted from calorie intake. ‘Obesity’ results, over time, from calorie intake exceeding the metabolic expenditure of calories. This model is compatible with a range of putative causes of ‘obesity’: it is possible that genetic, epigenetic, or social causes of ‘obesity’ generate their effect via disruption of calorie balancing (for example, through a genetic or epigenetic influence on hunger levels, or social barriers to exercise).

The ‘calories in-calories out’ model is prevalent in norms and discourses around diet and weight loss that drive individual behaviour, as well as public health research and policy interventions. Mackert (2016) outlines the ways in which calories have become a recurring motif in daily life, suggesting that “when individuals approach supermarket shelves, grab an item and check its caloric content, the calorie has inscribed itself into their routines”. Mackert (ibid.) traces the historical development of the calorie, from the operationalization of the calorie for food reform in the US by the chemist Wilbur Atwater in the 1880s through construction of human calorimeters: “that [the calorimeter] was now applied to human motors shows that the emergence of the calorie was both the effect and means of establishing a thermodynamic model of bodies”. This model has persisted: diets that aim to induce weight loss often rely on reduced calorie intake, or a calculation that subtracts calorie expenditure from calorie intake (Finer, 2001), and calorie counting apps such as Lose It!, MyFitnessPal, and Cronometer, remain popular.

In addition, public health researchers have turned their focus to ways to reduce caloric intake or increase caloric expenditure on a population level, in order to tackle the “obesity epidemic” (Howell & Kones, 2017; Kumanyika, 2008). This focus is reflected in policy interventions such as requirements for calorie labelling. In 2022, new calorie labelling regulations were implemented in England, requiring businesses over a certain size to provide calorie information on their menus, hailed as an “important milestone in government obesity policy” (Kaur et al., 2022).

For this model to be generally applicable as a proximate cause or explanation for body size (and therefore useful for public health interventions), it relies upon the assumptions that the metabolic conversion of food into energy can be precisely quantified, and that metabolism is “a laboratory whose working is basically the same for everyone” (Landecker, 2011, p. 173). Developments within postgenomic science have complicated this understanding. Landecker (2011) has analysed the shift in understandings of metabolism from within nutritional epigenetics, where a picture emerges of metabolisms that vary between individuals, shaped by and reactive to environmental (including social and maternal) context.

A shift away from an understanding of metabolism as a calculable process of energy conversion can also be seen in the postgenomic field of metabolomics. Metabolomics has been defined as the “comprehensive study of the metabolome, the repertoire of biochemicals (or small molecules) present in cells, tissues, and body fluids” (Beger et al., 2016, p. 148). This involves a patchwork of methods in order produce a metabolic profile of cells, tissues or organisms. Technological advances over the past 15 years have resulted in a significant increase in analytical capability, with the ability to quantify hundreds to thousands of metabolites in a biological sample in a small time frame, and enable metabolic phenotyping (ibid.). An individual’s metabolic state is taken as a close representation of their overall health status, in that the metabolic state reflects their genome and microbiome, as well as diet and other environmental factors. Clish (2015, p. 3) describes metabolomics as “an objective lens to view the complex nature of how physiology is linked to external events and conditions, as well as measure its response to perturbations such as those associated with disease”. Beger et al. (2016, p. 149) emphasise that “the metabolome is dynamic and rich, arising only partially as the product of our own gene-encoded proteins. It also arises from the metabolic products of the microbes within us, the air we breathe, the food we eat, and the water we drink”.

Metabolomics has not thus far received the level of attention that postgenomic sciences such as epigenetics or microbiome research have from philosophers or science studies scholars^3^. However, research within metabolomics provides a window into changing understandings of metabolism, and has important implications for understandings of ‘obesity’. The precision of metabolomics allows for an increasingly complex and dynamic picture of metabolism. Appreciation of the dynamic regulatory action of the metabolic interface appears to further disrupt the idea of metabolism as a formula that converts input to output, but is rather an ongoing set of heterogeneous processes of regulation, renegotiation, and integration. The technological advances of metabolomics allow for a molecular window into these processes, whereby “metabolism no longer is seen as a factory whose main job is to make bodily building blocks and create energy for movement and thought but has become a zone of regulation, a space for the integration and coordination of external and internal signals, whose timing is as important as their nature” (Landecker, 2013, p. 511).

Metabolomics technologies can also elucidate, or make legible, complex relationships or interactions: for example, a metabolic profiling study carried out by Koeth et al. (2013) showed that elevated plasma levels of trimethylamineN-oxide are at the nexus of a relationship between diet, gut microbiome composition, and risk for cardiac events. Additionally, metabolomics has the promise of applications in the realm of human health and disease, and has been heralded as a crucial tool for precision medicine (Clish, 2015; Beger et al., 2016; Schmidt et al., 2021). ‘Obesity’ in particular has arisen as a key target for metabolomics approaches. Metabolomics technologies have been used in within the DOHaD framework to understand the impact of the in utero environment on metabolic processes (Hivert et al., 2015; Safi-Stibler et al., 2020), or in conjunction with metagenomics technologies to understand the interplay between metabolism and the microbiome (Daliri et al., 2017).

I argue that, despite the potential for findings from metabolomics to emphasise dynamism and heterogeneity, deterministic assumptions nevertheless structure metabolomic research into ‘obesity’. This arises in the ways that the environment is characterised in metabolomics studies, the kinds of interventions that are introduced, and the attempt to determine a ‘metabolic signature’ of ‘obesity’ that reveals a persistent linking of ‘obesity’ and dysfunction.

## Fragmented environments in metabolomics

The fragmentation and reduction of the environment in postgenomic science has been explored by many scholars. In the context of environmental epigenetics research, critics have highlighted the ways in which the conceptualisation of the environment in this area are reductionist and exclude social complexity, often restricting the environment to the maternal *in utero* enviroment or viewing features of the environment such as nutrition in isolation from wider structural factors (Richardson, 2015, 2021; Müller et al., 2017). In microbiome research, researchers have argued that complex social categories or practices are reduced to isolated or molecularised environmental influences, and that characterisations of the social environments of racialized groups are limited or distorted (Benezra, 2020; Nieves Delgado & Baedke, 2021).

Many studies in metabolomics exhibit similar biases to those noted by Müller et al. (2017) in their discussion of childhood ‘obesity’, in that they focus on narrow individualised interventions. Metabolomics studies that aim to characterize the effect of an intervention on the metabolome often focus on the effect of dietary or nutritional interventions, and sometimes the impact of particular supplements in isolation (Nieman et al. 2012; Gu et al., 2013; Kim et al., 2013; Romo-Hualde et al., 2018). Some studies use metabolomics techniques to measure changes in metabolic profile after exercise interventions (Schranner et al., 2020; Kelly et al., 2020), and the effect of bariatric surgery (Liu et al., 2017). The studied dietary and exercise interventions are typically short to medium term, occurring over a span of weeks or months: in Handakas et al’s (2022) systematic review of metabolomic studies of childhood ‘obesity’, the longest intervention period was one year, and the shortest was three weeks. The interventions consisted of dietary changes (either imposed through participants being physically located at a camp for the duration of the intervention, or through provision of dietary guidelines), exercise regimes, and (in some studies) nutrition or weight loss education.

The focus on specific dietary or exercise changes indicates a narrow construal of the features of the environment which are taken to have an effect on metabolism. I suggest that this narrow focus results in a distorted picture of the relationship between the environment and the body’s metabolism, whereby only some aspects of the environment are investigated as the primary influence on metabolic processes. Despite the promise of metabolomics to further uncover the dynamic regulatory action of the metabolic interface, this appears to reflect the persistence of the ‘calories in, calories out’ model, in terms of the sustained attention to effects of calorie intake and calorie expenditure.

The apparent advantage of metabolomics is that it can provide an integrated picture of the metabolic entanglements with a variety of different entities or processes, including the genome, microbiome, and the environment. However, this is at odds with the narrow understanding of the environment that is at work in metabolomics research. One aspect of the environment that is comparatively neglected is the social environment. This may be particularly important in the context of the application of metabolomics to the question of ‘obesity’ or fatness. The existence of fatphobia or weight discrimination is well-documented: empirical evidence suggests that there are several ways in which fat people have systematically different life experiences, and therefore social environments, compared to others. This includes differences in income level, differences in access to and quality of healthcare, mental health, and workplace discrimination (Rubino et al., 2020). There are many ways in which these differences in social environment might have an impact on metabolism. For example, there is evidence that psychosocial stress can influence metabolic processes (Sanghez et al., 2013; Mellon et al., 2018). We might expect that pervasive institutional and interpersonal fat phobia or weight discrimination results in increased levels of psychosocial stress for those who are the target of this, in a similar way to research which shows that racial discrimination can have physiological effects (Goosby & Heidbrink, 2013; Dolezsar et al., 2014; Currie et al., 2019).

Additionally, particularly in studies that look at interventions over the span of months or a year or those that look at bariatric surgery patients, reduction in weight stigma and changes in the social environment of participants may be a confounding factor. Sustained weight loss can involve not only a change in body size but also a corresponding change in the level of weight stigma individuals experience. One indication that weight loss may result in reduced weight stigma comes from a study where participants viewed an image of an ‘obese’ woman and were asked to indicate their perceptions of the woman’s behaviours and personality characteristics (Fardouly & Vartanian, 2012). They were then shown a more recent image of the woman and were informed that she had lost weight. Participants judged that the woman was eating more healthily, exercising more often, and perceived her as more ‘competent’ and less ‘sloppy’ after losing weight. While weight stigma may not entirely disappear for individuals who have lost weight, a reduction in weight stigma could mean a reduction in (for example) psychosocial stress. If the effects of weight stigma can affect the metabolome, such as through the pathway of psychosocial stress, then observed changes in the metabolome following a weight loss intervention may not be solely due to the intervention itself, but rather changes in the individual’s membership in a stigmatized group.

A related consideration arises in some of the studies included in Handakas et al’s (2022) review of metabolomics and childhood ‘obesity’, which looked at a yearlong intervention for ‘obese’ children that included a series of counselling and education sessions for both the children and their parents. The harms of perpetration of weight stigma by family members is well documented (Himmelstein & Puhl, 2019; Roberts et al., 2021; Lawrence et al., 2022). To the extent that parental counselling and education reduced stigmatising interactions with family members, this may have also had an effect on the metabolome through a reduction in psychosocial stress. This would be a change that does not operate via the causal pathway of weight loss in itself. However, this cannot be determined from the existing studies, as measures of stigma were not obtained.

The neglect of the social environment reveals a tension between stated understandings of the metabolome as providing a snapshot of the health state of an individual, which reflects the range of causal influences, and metabolomics research which restricts the features of the environment which are characterised and investigated. If the goal of the use of metabolomics is to understand patterns of metabolism across body sizes, it is necessary to take into account features of the social environment that could be causally relevant.

I have suggested that a constrained and fragmented picture of the environment appears to be in operation in metabolomics research, where particular features of the environment (dietary intake and exercise levels) are afforded an outsized causal, even determinative, role, and other potentially causally relevant features (notably, structural and interpersonal fatphobia that leads to fat people having systematically different social environments) are neglected almost entirely. This results in the features of the environment that are investigated, dietary intake and exercise levels, appearing to determine, to a significant extent, metabolic states or patterns. This is suggestive of the persistence of the calories in, calories out model of determining body size. The focus on discovering effective weight loss interventions rather than the features of the environment that negatively impact fat people is also reflective of the commitment to the biomedical model of ‘obesity’, where body size determines health status. The ways in which the biomedical model, and its presupposition of ‘obesity’ as pathology, are also evident in choices made in metabolomics research, will be expanded upon in the following section.

## A metabolic signature of ‘obesity’ and the persistent determinism of the connection between ‘obesity’ and disease

There has been a raft of studies that aim to determine a ‘metabolic signature’ of ‘obesity’ (Rangel-Huerta et al., 2019 provides a review). The goal of this research is to identify specific metabolic patterns that are associated with ‘obesity’, which could then be used as biomarkers for use in clinical settings. I argue that this goal is complicated by the existence of significant metabolic heterogeneity across body sizes. The commitment to the deterministic connection between ‘obesity’ and pathology or disease are illustrated by, firstly, flexibility in the use of ‘obesity’ to mean body size or dysfunction, and, secondly, the way that patterns of metabolic heterogeneity are classified and organized.

Research into a metabolic signature of ‘obesity’ (MSO) aims to identify differences in metabolic states between ‘obese’ and ‘normal weight’ individuals, thereby providing a characterization of a constellation of metabolic markers that are associated with ‘obesity’. Comparisons have been carried out in various populations, including adult men, pregnant women, and children (Yu et al., 2018; Houttu et al., 2018; Butte et al., 2015). Other studies investigate associations between ‘obese’ metabolic signatures and diseases such as diabetes, asthma, and cancer (Pan et al., 2022; Liu et al., 2018; Kliemann et al., 2021). Discovery of altered or dysfunctional metabolic states associated with ‘obese’ individuals could shine light upon the mechanisms by which ‘obesity’ is connected to negative health outcomes.

However, whilst correlations have been found between metabolic states and body size, there is also evidence of significant heterogeneity in terms of metabolic state (or levels of metabolic function or dysfunction) within body size categories. This has been a known phenomenon since the 1980s: Ruderman et al. (1981) first identified the existence of what they termed “metabolically obese, normal weight” individual. These are individuals who are classified as ‘normal weight’ by BMI, and also exhibit some indicators of metabolic dysfunction, such as hyperinsulinism and an increase in fat cell size. Ruderman and colleagues challenged the notion that standard weight–height tables (this was prior to the dominance of the BMI as a measure of body size) were the proper way to determine high-risk groups for so-called obesity associated disorders. They observed normal weight individuals exhibiting type 2 diabetes, coronary heart disease, hypertriglyceridemia, and hyperinsulinemia, and noticed that these conditions thought to indicate metabolic abnormalities could not be explained by skinfold thickness or adipose mass.

Later research constructed further categories of the intersection between metabolic state and body size. In addition to the ‘metabolically healthy obese’, additional categories of ‘metabolically obese normal weight’ (those who are classified as ‘normal weight’ by BMI and exhibit indicators of metabolic dysfunction, such as low insulin sensitivity, high liver fat, and high triglycerides), the ‘“at risk” obese’ or ‘metabolically unhealthy obese’ (those who are classified as ‘obese’ by BMI and exhibit indicators of metabolic dysfunction) and the ‘metabolically healthy individual’ (those who are classified as ‘normal weight’ by BMI and exhibit indicators of good metabolic function, including high insulin sensitivity, low liver fat, and low triglycerides) have been proposed (Karelis et al., 2004). Additional categories have subsequently been introduced such as that of ‘normal weight obesity’, a category characterised by higher levels of body fat at a ‘normal’ BMI, of which ‘metabolically obese normal weight’ individuals are taken to be a subset (De Lorenzo et al., 2006; Oliveros et al., 2014).

Studies have produced varying estimates of the prevalence of ‘metabolically healthy obese’ individuals in the population, ranging from 10 to 51% of ‘obese’ individuals (Rey‐López et al., 2014 provides a review). Estimates of the prevalence of ‘metabolically obese normal weight’ individuals similarly varies, with one systematic review finding a range of 12.9 to 23.5% of ‘normal weight’ individuals (Gómez-Zorita et al., 2021). Romero-Corral et al. (2010) found that 23.1% of American men and 33.3% of American women are classified as having ‘normal weight obesity’, and estimate that 30 million Americans fall into this category. Part of the variation in the estimates produced stems from variation in the definitions of these categories. However, even the lower end of these estimates is suggestive of meaningful levels of heterogeneity in metabolic state across body sizes.

Despite longstanding evidence for metabolic heterogeneity, MSO studies often do not acknowledge this heterogeneity or account for the ‘metabolically healthy obese’ or ‘metabolically obese normal weight’ subtypes within their study designs. In one systematic review of 60 MSO studies, only 6 studies recognized the existence of ‘metabolically healthy obese’ individuals (Rangel-Huerta et al., 2019). These 6 studies were all investigations of the ‘metabolically healthy obese’ type in themselves. That is, studies that did not have the goal of explicitly investigating the metabolome in ‘metabolically healthy obese’ did not consider the existence of ‘metabolically healthy obese’ individuals as problematic or in need of acknowledgement when determining the metabolic signature of ‘obesity’ per se. Not only are ‘metabolically healthy obese’ individuals ignored, they are sometimes explicitly understood as an anomaly to be separated out from analysis. Karelis et al. (2004) suggest that the inclusion of ‘metabolically healthy obese’ individuals in the same cohort as ‘at risk obese’ individuals, and ‘metabolically obese normal weight individuals’ in the same cohort as ‘lean individuals’, in clinical research could cause problems with data interpretation, and indicate that these groups should be analysed separately. However, the exclusion of these individuals, in, for example, a trial comparing the health outcomes of ‘obese’ and ‘lean’ participants, would, by design, increase the likelihood of associating fatness with negative health outcomes or metabolic dysfunction.

### Meanings and classification

In addition to the marginalization of metabolic heterogeneity in MSO research, the way in which metabolic heterogeneity itself has been investigated demonstrates the extent to which the deterministic relationship between ‘obesity’ and pathology or dysfunction is deeply entrenched as an organising principle. I argue that this appears in the flexible use of the concept of ‘obesity’, which can sometimes refer to body size and sometimes to dysfunction, and in the practices of classification.

Within MSO studies, ‘obesity’ is defined in terms of body size for the purposes of categorising participants into ‘obese’ or ‘lean’ groups or as a variable against which to measuring associations with metabolic markers. The standard measure used is BMI, sometimes in conjunction with other measures such as waist circumference or waist-to-height ratio, This is ubiquitous: in Rangel-Huerta and colleagues’ (2019) systematic review of 60 MSO studies, all 60 studies used BMI as at least one measure of ‘obesity’. The concept of ‘obesity’ as defined by body size or BMI is also frequently part of the framing of the significance of MSO research. In the same systematic review, 41 out of 60 studies made reference to the ‘obesity epidemic’ or ‘obesity’ as a global health crisis, relying on epidemiological studies that define ‘obesity’ in terms of BMI.

However, when metabolic heterogeneity is considered, the meaning of ‘obese’ can shift: it can refer to metabolic dysfunction, regardless of body size. Two concepts of ‘obesity’ are in operation: on the one hand, ‘obesity’ is typically defined by body size (typically BMI). This is how individuals are classified as ‘obese’ or ‘normal weight’ for the purposes of comparison of metabolic states, as well as how the category is used in the clinic and often in public discourse around fatness. On the other hand, ‘obesity’ is sometimes used as a term synonymous with disease or dysfunction, as evidenced by the terms ‘metabolically obese’ and ‘normal weight obesity’. Even within the classification of metabolic heterogeneity, ‘obese’ takes on two meanings: for the category ‘metabolically obese normal weight’, ‘obese’ means dysfunctional, and for the category ‘metabolically healthy obese’, ‘obese’ means larger body size (higher BMI). This slippage between the two meanings indicates the ongoing commitment to fatness as abnormal or dysfunctional: larger bodies are marked out by the medicalized term ‘obesity’, whether or not they exhibit disease or dysfunction, and disease or dysfunction even in the absence of larger body size becomes ‘obese’.

Another indication of this deterministic commitment is evident in the creation of the categories themselves. Why, when faced with metabolic heterogeneity across bodies of all sizes, did researchers create a taxonomy that split ‘obese’ and ‘lean’ individuals by metabolic function? A close examination of the development and uptake of these categories is outside the scope of this paper. However, ongoing disputes over how to define these categories that result in varying estimates of prevalence (Gómez-Zorita et al., 2021) indicate that there is no self-evident or uncontroversial way of dividing populations into the categories ‘metabolically healthy obese’, ‘metabolically unhealthy obese’, ‘metabolically obese normal weight’, and ‘metabolically healthy’.

Philosophers have argued for the ways in which values inevitably enter into practices of scientific classification, sometimes legitimately and sometimes in ways that systematically distort understanding (Conix, 2020; Reydon & Ereshefsky, 2022; Intemann, 2001). Anderson (1995) argues that the way in which the complexity and messiness of the world precludes the existence of only one epistemically significant system of classification. Non-epistemic (social, political, or moral) values therefore must enter into choices of which classificatory scheme to use, and Anderson highlights ways in which androcentric or sexist values can enter into classifications in economic and psychological research. In the classification of metabolic heterogeneity, the commitment to retaining the differentiation between ‘obese’ and ‘lean’ bodies could be a reflection of the broader social commitment to ‘obesity’ as diseased, unhealthy, or dysfunctional, which is linked to moralizing and stigmatizing attitudes about fatness.

One alternative to the current classification would be to embrace the complexity and indeterminacy of patterns of metabolic heterogeneity. This would mean a move away from body size or BMI as an organizing principle for classificatory schemes and towards a mapping of continuous patterns of metabolic processes that explores interactions and associations with a range of factors, without being tightly coupled to body size. This is a live possibility within metabolomics research: some studies have identified biomarkers of ‘metabolic wellness’ independently of body size or BMI (Batch et al., 2013; Wiklund et al., 2014).

## Conclusion

‘Obesity’ continues to be framed as an epidemic or pandemic that requires scientific attention and the urgent development of biomedical interventions, and ‘obesity’ persists as a key target within postgenomic science. Whilst the conceptual shifts within fields of postgenomic science such as epigenetics presents a challenge to genetic determinism, forms of environmental determinism have emerged (Richardson, 2015; Lock, 2015; Waggoner & Uller, 2015; Meloni et al, 2022). I have examined research into ‘obesity’ in the field of metabolomics to argue that, despite the potential for metabolomics to inform an understanding of metabolism as dynamic, complex, and interactive, determinism appears within metabolomic science. I have argued that a form of environmental determinism can be found in the fragmentation and reduction of the environment in metabolomic investigations of interventions, and the neglect of the social environment. I have also argued that another form of determinism persists in metabolomics: the deterministic connection between ‘obesity’ and dysfunction. This is demonstrated by the ways in which metabolic heterogeneity is dealt with, through its marginalization in scientific investigations, the flexible meaning of ‘obese’ in metabolic classifications, and the construction of the classifications themselves.

As postgenomic science has developed, the potential harms of postgenomic determinism have been brought to light. These harms include reinforcing sex or racial essentialism, marking out racialized and classed populations as biologically disadvantaged, and placing individual responsibility onto (often racialized) mothers (Richardson, 2017; Benezra, 2020; Kenney & Muller, 2017; Valdez, 2021; Saldaña-Tejeda & Wade, 2019). Whilst determinism in postgenomic investigations of the role of the maternal environment in producing ‘obesity’ bears the risk of increasing the burden of responsibility on mothers of ‘obese’ children, the normative implications of environmental determinism for ‘obese’ individuals themselves is complex. The language of choice and responsibility has been a defining feature of discourses around fatness. Weight stigma or fatphobia is often fuelled by the belief that fat people have the ability to lose weight (Puhl & Heuer, 2010). Given that they apparently have this agency, weight stigma becomes justified, either as a deserved consequence of an individual choice to be fat, or even as a tool to attempt to induce weight loss. In contrast, the deterministic narratives (whether they be genetic or environmental), that position fatness as outside of an individual’s control have the potential to challenge this assumption and the associated stigma.

Despite this, the forms of determinism I have identified within metabolomics do come with risks to fat people as a marginalized group. The fragmentation of the environment and failure to take into account the wider social environment within metabolomics contribute to the neglect of weight stigma, discrimination, and structural inequality as active pathways that affect biological processes. As fat studies scholars have noted, health justice is not possible without the recognition of the harms to health of fat people as a consequence of stigma, discrimination, inequality, and oppression (Pausé et al., 2021; Rich et al., 2010; Mollow, 2015). Investigation of the effects of weight stigma on the metabolome, as well as on epigenetic modifications and the microbiome, could illuminate the ways in which this kind of social inequality interacts with biological processes and possibly affects health outcomes.

Additionally, the deterministic connection between ‘obesity’ and dysfunction can reinforce stigmatizing and shaming attitudes towards fat people, and can provide legitimation for efforts to induce weight loss on either an individual or population level regardless of the costs (Saguy & Riley, 2005). This form of determinism emerged long before the advent of postgenomic science (Strings, 2019). However, the ‘translational imperative’ of postgenomic science orients scientific efforts towards the fast and effective development of interventions (Huss, 2014), and in the case of ‘obesity’, this often means a drive to translate postgenomic research into weight loss treatments. Harms can arise from the development and marketing of weight loss interventions premised upon weight loss as a necessary cure for ‘obesity’ conceived as necessarily diseased and dysfunctional.

Metabolomics is one area of ‘obesity’ science where deterministic assumptions structure inquiry in ways that potentially contribute to fat oppression. These assumptions also appear to arise in part from broader social attitudes towards fatness. Future research exploring the investigation of ‘obesity’ in a range of scientific fields could shed light on the interplay between weight stigma and fat oppression, social and political values, assumptions and choices within ‘obesity’ science, and construction of ‘obesity’ or fatness that emerges from this science.

## Footnotes

1 I follow Lewontin (1996) and Kitcher (2001) in characterising genetic determinism as the thesis that genetic causes take priority in the explanations of traits, leading to claims of innate difference or innate capacity. Acknowledgement of the role of genes in the context of their interaction with other factors does not constitute genetic determinism, in my view.

2 It should be noted that fatness is both racialized and gendered, and that recent scholarship has highlighted intimate connections between anti-Blackness and anti-fatness in particular (Shackelford, 2021; Harrison, 2021; Strings, 2019). Although analysis of the intersection between race and fatness is beyond the scope of this paper, the ways that fatness is racialized may provide further nuance to the ways in which determinism emerges within postgenomic science and how it is taken up within the public sphere.

3 Although, it should be noted that Hannah Landecker’s (2011, 2013, 2019) work on changing understandings of metabolism is an important example.
